# Age- and sex-dependent associations between self-reported physical activity levels and self-reported cardiovascular risk factors: a population-based cross-sectional survey

**DOI:** 10.1186/s12889-024-20351-w

**Published:** 2024-10-16

**Authors:** Johannes Burtscher, Martin Kopp, Jeannette Klimont, Hanno Ulmer, Barbara Strasser, Martin Burtscher

**Affiliations:** 1 Institute of Sport Sciences, University of Lausanne, Lausanne, CH-1015 Switzerland; 2 Institute of Sport Science, University of Innsbruck, Innsbruck, A-6020 Austria; 3 Unit Demography and Health, Directorate Social Statistics, Vienna, 1110 Statistics Austria Austria; 4 Institute of Medical Statistics and Informatics, Medical University of Innsbruck, Innsbruck, 6020 Austria; 5 Ludwig Boltzmann Institute for Rehabilitation Research, Vienna, 1100 Austria; 6 Faculty of Medicine, Sigmund Freud Private University, Vienna, 1020 Austria

**Keywords:** Exercise, Energy expenditure, Health, Disease, Life-style, Aging, Gender

## Abstract

**Background:**

The amount of regular physical activity (PA) can modulate the prevalence of traditional risk factors for cardiovascular disease (CVD) such as obesity, systemic hypertension, hypercholesterolemia, and type 2 diabetes (T2D). However, how different PA levels either below (< 600 MET min/week), within (600–1200 MET min/week), or above (> 1200 MET min/week) the range of the minimal WHO recommendations impact the age- and sex-dependent prevalence of these risk factors remains to be elucidated.

**Methods:**

This cross-sectional study was performed to evaluate these relationships using population-based self-reported data collected in a central European country (Austria, 2019). The sample included a total of 15,461 persons (7166 males: 16–95 + years, BMI 26.6 ± 4.4; 8295 females: 16–95 + years, BMI 25.1 ± 5.0). Besides various lifestyle factors (e.g., dietary habits, smoking, and alcohol consumption), variables of particular interest were the age- and sex-dependent amount of weekly PA and prevalence of risk factors for CVD. Sex-specific logistic regression analyses were applied to estimate adjusted odds ratios (ORs) for the associations between self-reported PA and risk factor prevalence.

**Results:**

Relatively small beneficial effects were found regarding the prevalence of risk factors for CVD when achieving PA levels corresponding to 600–1200 MET min/week as compared to those who did not meet these recommendations. However, exceeding the WHO recommendations provided much more pronounced benefits, especially in younger and older age groups. Adjusted ORs revealed that high volumes of PA (> 1200 MET min/week) were associated with a 32–43% reduction in the prevalence of obesity and T2D compared to those who did not achieve the WHO recommendations (< 600 MET min/week), as well as with a lower prevalence of systemic hypertension only in women and a lower prevalence of hypercholesterolemia only in men.

**Conclusions:**

Exceeding minimal WHO recommendations for PA promises large beneficial effects, particularly on the prevalence of obesity and T2D. Demonstrated sex differences in PA levels and their association with cardiovascular risk factors may provide an important basis for preventive health counseling.

## Background

The 2020 World Health Organization (WHO) guidelines on physical activity (PA) for adults recommend 150–300 min of moderate intensity or 75–150 min of vigorous intensity PA per week or more [1]. However, greater PA-related health and longevity benefits may be obtained in a dose-dependent manner by PA amounts that exceed the recommended minimum by 3 to 5 times or higher [2]. Although life expectancy seems to increase steadily as moderate-intensity PA increases, at least up to an amount of 100 min per day [3], differential sex- and-age-dependent PA effects on traditional cardiovascular risk factors such as obesity, systemic hypertension, hypercholesterolemia, and type 2 diabetes (T2D) are less clear. Generally, all these risk factors can be reduced by PA to various degrees, depending on the mode, intensity, and volume of the PA performed. Already achieving minimal PA recommendations can reduce visceral adipose tissue [4], which increases with age, but more in males than in females and regardless of body composition [5]. Furthermore, altering a sedentary lifestyle to include the recommended minimum of 150 min of moderate-intensity aerobic activity per week was associated with a 26% lower T2D risk [6]. PA volumes exceeding the WHO recommendations were particularly effective in reducing obesity and T2D prevalence [7]. Most prospective studies have demonstrated a rather modest PA effect on systemic blood pressure in both non-hypertensive and hypertensive individuals, but a linear relationship between PA volume and lowering of blood pressure also seems to exist [8]. Similarly, relatively small improvements in blood lipids following PA intervention have been reported, namely an increase in high-density lipoprotein cholesterol (HDL-C) and a decrease in low-density lipoprotein cholesterol (LDL-C) and triglycerides (TG) [9]. The relationship between the amount of PA and these beneficial effects might also be linear [10], although a more recent study found a linear risk reduction in cardiovascular disease markers up to a step-count of 10,000 per day [11]. Although some age-dependent and sex-specific differences of PA-related benefits on traditional cardiovascular risk factors have been reported [12], more information about sex- and age-dependent associations across different PA levels is needed and, specifically, below, within, and above the range of the minimal WHO recommendation. Unhealthy lifestyles and behavioral risk factors remain important drivers of mortality in Austria. About 36% of deaths in 2019 could be attributed to smoking, dietary risks, alcohol, and low levels of PA (OECD/European Observatory on Health Systems and Policies, 2023), suggesting room to improve prevention and healthcare, including the importance of addressing PA and nutrition recommendations for disability prevention. Recent results from the Austrian Health Interview Survey (ATHIS) revealed a rising trend in the prevalence of disability among older adults in Austria with a strong positive association between PA participation and functional mobility, especially in women [13]. This cross-sectional study was carried out to evaluate the relationship between PA levels and cardiovascular risk factors using population-based data in a central European country (Austria).

## Methods

The modulation of traditional risk factors (i.e., obesity, hypertension, hypercholesterolemia, and T2D) by PA was evaluated on the basis of data collected in the ATHIS 2019 [14].

Sample: The ATHIS sample is a sample of individuals. The basis for drawing the sample was the Central Register of Residents (ZMR). For reasons of better representativeness, the gross sample was spatially stratified. The stratification was based on the 32 health care regions as defined in the Austrian Health Care Structure Plan. Assuming a 48% response rate, this resulted in an Austria-wide gross sample of 32,101 people. Of these, 1,493 addresses were qualified as neutral drop-outs at address level (“address exists, no contact possible”, “building/apartment vacant”, “target person not present at address during field period or secondary residence”, “no private household or building site”). This means that 30,608 persons formed the basis for the participation rate. A total of 15,461 people answered the health survey (net sample). The nationwide response rate was 50.5%.

Imputation: Imputation was carried out in the case of item non-response, i.e. when information was missing for a single characteristic. For most answers, there were only a few refusals or “I don’t know” responses. Only in the case of the question on monthly household income was there no response for 8.6%. In proxy surveys, an imputation of the complete questionnaire was carried out based on the response behavior in the shortened questionnaire. Imputation was carried out using the KNN (k-Nearest Neighbor) method, whereby the distance variables for each variable to be imputed were specified after a basic analysis of the item non-response. The following variables were always included in the calculation of the distance function: gender, age, education and region of care. Furthermore, the variables subjective state of health, chronic illness (yes/no), restrictions in everyday activities and pain were also considered in the imputation of selected variables.

A case distinction was made for the missing values for the variable “exact income”, as the “income in categories” was known for half of the cases. This could then be used as an additional distance variable with a very high weighting. As an “I don’t know” response is an important statement in the case of vaccinations, no imputation was made in these cases.

Sample weights: In a random sample, a reduced image of the population is created. When calculating the statistical results, this reduced selection then serves as the starting point for the representation of the population, which is carried out by means of so-called extrapolation. In this process, the characteristic values collected with the help of the sample are used to estimate the parameters of interest in the population. The extrapolation (weighting) of the data was carried out in several steps. The first step was to determine the base weight, which corresponds to the reciprocal value of the selection probability of a household in the microcensus. The non-response adjustment was then carried out using a logistic model, whereby variables from the selection frame could be used for that. Finally, weights were calibrated using known key figures from the population.

PA and lifestyle factors: Besides various lifestyle factors (e.g., dietary habits, smoking, and alcohol consumption), variables of particular interest were the amount of weekly PA (as exposure) and the prevalence of obesity, hypertension, hypercholesterolemia, and T2D (as outcomes).

The ATHIS data are self-reported, and the body mass index (BMI) was calculated by dividing the reported body mass in kilograms by height in meters squared. Obesity was defined as a BMI of 30 or higher, and the presence of other risk factors (hypertension, hypercholesterolemia, and T2D) was also self-reported when based on a medical diagnosis made in the previous 12 months. In order to carry out a rough evaluation of the potential impact of lifestyle factors, the following categorization was chosen based on self-reported information for a typical week: the frequency and/or amount of smoking (i.e., daily vs. seldom or not), alcohol consumption (i.e., on more than two days vs. on less than 3 days), and dietary intake (i.e., daily fruit and vegetable consumption vs. less than daily, meat or fish consumption on more than two days vs. on less than 3 days).Respondents reported the volume (minutes) on active commuting (e.g., walking or biking) and that on sports and recreational PA (e.g., hiking, biking, jogging, swimming, etc.) in a typical week. From these data, the individual PA-related energy expenditure was calculated and reported in metabolic equivalent (MET) minutes as a multiple of the resting energy expenditure (= 1 MET, corresponding to 3.5 mL oxygen consumption per minute per kg body mass). As an example, walking, hiking, or jogging at a pace requiring 4 METs per hour (60 min) is equal to 240 MET minutes. Based on the WHO guidelines that recommend a weekly minimum amount of PA of 150–300 min at moderate intensity (4 METs) [7], we subdivided the study population into three categories: (1) < 600 MET min/week (not reaching WHO recommendations), (2) 600–1200 MET min/week (following minimum WHO recommendations, and (3) > 1200 MET min/week (exceeding minimum PA WHO recommendations). In addition, 3 age categories were compared; 15–44 years (reflecting early to middle age), 45–65 years (late middle age), and ≥ 65 years (after retirement, seniors).

### Statistics

For this study, publicly available data from ATHIS provided by Statistics Austria were used. For sample selection, participation rate, the use of sampling weights, and information on handling of missing values by imputation, see [14]. Characteristics of the study participants and self-reported risk factor prevalence were tabulated by age, sex, and PA using means and ranges for continuous variables and percentages for categorical variables. Confidence intervals (95% CI) for proportions and chi-square tests were calculated to evaluate differences in risk factor prevalence between the different groups. Due to the multiple comparisons and the fact that the presented age groups were chosen arbitrarily, *p*-values should be interpreted in an explorative way only. Sex-specific logistic regression analyses were used to estimate adjusted odds ratios (OR) and their 95% CIs for associations between self-reported PA and risk factor prevalence, self-reported obesity, hypertension, hypercholesterolemia, and T2D. In a first step, adjustments were performed for age, smoking, alcohol consumption, and four key variables concerning dietary intake (fruit, vegetable, meat, and fish intake). In a second step, models for hypertension, hypercholesterolemia, and T2D were additionally adjusted for BMI. Statistical analyses were performed with MedCalc Software Ltd. (https://www.medcalc.org/calc/, version 22.016; accessed January 1, 2024) and IBM SPSS version 26.0 (IBM SPSS Statistics for Windows, Chicago, IL, 123 USA). *P*-values (2-sided) below 0.05 were considered as indicating statistical significance.

## Results

Characteristics of the study population by sex are shown in Table 1. Men were more physically active than women, but more often smoked regularly (20.5% vs. 16.6%), drank alcohol more often (28.7% vs. 8.9%, on two or more days/week), and consumed fewer fruits and vegetables but more meat.

**Table 1:** Characteristics of the male and female study populations

Characteristic	Frequency (%) range, mean (SD)	
Sex *N* (total: 15,461)	Male 7166 (46)	Female 8295 (54)
Age (range)	16–95+	16–95+
Height (cm)	177.8 (6.8)	164.8 (6.2)
Body mass (kg)	84.0 (14.8)	68.2 (13.6)
BMI kg/m^2^	26.6 (4.4)	25.1 (5.0)
Physical activity		
< 600 MET min/week 600–1200 MET min/week > 1200 MET min/week	3509 (49.0) 1429 (19.9) 2228 (31.1)	4384 (52.9)* 1828 (22.0)* 2083 (25.1)*
Smoker (daily)	1466 (20.5)	1381 (16.6)*
Alcohol consumption (on more than two days/week)	2057 (28.7)	735 (8.9)*
Fruit consumption (daily)	2995 (41.8)	4957 (59.8)*
Vegetable consumption (daily)	2877 (40.1)	4698 (56.6)*
Meat consumption (on more than two days/week)	6018 (84.0)	5358 (64.6)*
Fish consumption (on more than two days/week)	331 (4.6)	392 (4.7)

* indicates significant differences between sexes (*p* < 0.05)

The age- and sex-stratified prevalence of risk factors is depicted in Table 2. The weekly amount of PA increased until the age of 66–75 years for both sexes and was significantly higher in men in the age groups of 16–25, 46–55, and 66–95 years as compared to women. The prevalence of all risk factors (obesity, systemic hypertension, hypercholesterolemia, and T2D) increased with age for both sexes and was highest in the age group of 56–85 years. Obesity was more prevalent in men than women of 36–65 years of age. While systemic hypertension was more prevalent in men of 16–55 years of age, this prevalence was higher in women (57.2% vs. 49.2%) in the age group of 76–85 years. Hypercholesterolemia was more prevalent in women of 16–25 years of age (2.5% vs. 0.8%) but was more frequent in men of 36–55 years of age. The prevalence of T2D was higher in men compared to women in the age group of 46–75 years.

**Table 2:** Age- and sex-dependent amount of weekly physical activity and the prevalence of cardiovascular risk factors (obesity, hypertension, hypercholesterolemia, and diabetes)

Age group (years)	Sex	*n*	Physical activity (MET min/week)	BMI ≥ 30 (%)	Hypertension (%)	Hypercholesterolemia (%)	Diabetes (%)
16–25	Men Women	842 905	943 (1007) * 751 (788)	7.4 6.7	2.7 * 1.3	0.8 * 2.5	0.4 0.8
26–35	Men Women	931 1108	975 (1149) 940 (982)	13.7 11.1	3.8 * 2.1	6.3 5.2	0.6 0.5
36–45	Men Women	1010 1164	982 (1116) 917 (1010)	18.1 * 11.9	9.0 * 4.8	11.1 * 8.3	1.1 1.0
46–55	Men Women	1267 1436	1139 (1223) * 983 (1069)	20.7 * 16.9	21.2 * 15.1	23.5 * 15.4	4.3 * 2.7
56–65	Men Women	1318 1485	1275 (1314) 1215 (1273)	23.7 * 20.2	32.8 31.3	29.1 27.5	9.2 * 5.9
66–75	Men Women	960 1078	1463 (1560) * 1270 (1292)	22.7 22.6	47.1 45.5	30.6 33.9	18.1 * 12.8
76–85	Men Women	677 806	1122 (1285) * 782 (970)	17.4 19.4	49.2 * 57.2	31.0 35.7	16.2 16.1
86–95	Men Women	141 235	604 (989) * 325 (577)	8.5 10.6	51.1 60.9	24.8 31.1	15.6 15.7
> 95	Men Women	20 78	130 (307) 61 (182)	10.0 6.4	40.0 42.3	10.0 17.9	10.0 6.4

* indicates significant differences between sexes (*p* < 0.05)

Table 3 shows sex- and PA-dependent (< 600 MET min/week, 600–1200 MET min/week, and > 1200 MET min/week) risk factors for three age groups (16–45 years, 46–65 years, and older than 65 years). Within the youngest age group (16–45 years of age), only the prevalence of hyperlipidemia in men was lower in those who spent 600–1200 MET min/week than those who spend < 600 MET min/week. In the older age groups, prevalences of almost all risk factors increased markedly, but increasing PA levels partially counteracted this increase. The most pronounced PA effects were seen on the prevalence of obesity and T2D for both sexes. The T2D prevalence in men decreased from 22% in the group of the lowest PA level to 10% in the highest PA level and in women from 17.9–7.5%.

**Table 3:** Sex- and physical activity-dependent prevalences of cardiovascular risk factors for three age groups

Age (years) Sex	Physical activity < 600 MET min/week					Physical activity 600–1200 MET min/week					Physical activity > 1200 MET min/week				
	*n*	BMI ≥ 30 (%)	Hypertension (%)	Hypercholesterolemia (%)	Diabetes (%)	*n*	BMI ≥ 30 (%)	Hypertension (%)	Hypercholesterolemia (%)	Diabetes (%)	*n*	BMI ≥ 30 (%)	Hypertension (%)	Hypercholesterolemia (%)	Diabetes (%)
**16–45**															
Male	1517	15.0	5.3	7.4	0.8	539	13.2	5.0	4.1^a^	0.7	727	10.3	5.6	6.1	0.6
Female	1771	10.8	3.2	5.5	0.9	749	7.9	2.3	5.9	0.8	657	9.3	2.6	5.5	0.5
**46–65**															
Male	1168	24.6	29.3	30.7	7.9	527	22.8	27.1	24.5	5.7	890	18.9^a^	24.2	21.7^a^	6.0
Female	1408	22.5	26.1	22.0	5.1	649	17.6	22.7	22.3	4.3	864	13.0	19.4^a^	20.1	3.0
**> 65**															
Male	838	22.9	50.6	32.3	22.0	349	19.5	47.6	25.8	17.8	611	14.7^a^	45.0	29.5	10.1^a, b^
Female	1215	24.3	56.5	35.0	17.9	420	16.0^a^	50.0	31.2	12.1^a^	562	12.1^a^	41.3^a, b^	32.7	7.5^a^

^a^ means significantly different from PA < 600 and ^b^ from 600 to 1200 MET min/week

**Fig. 1:**
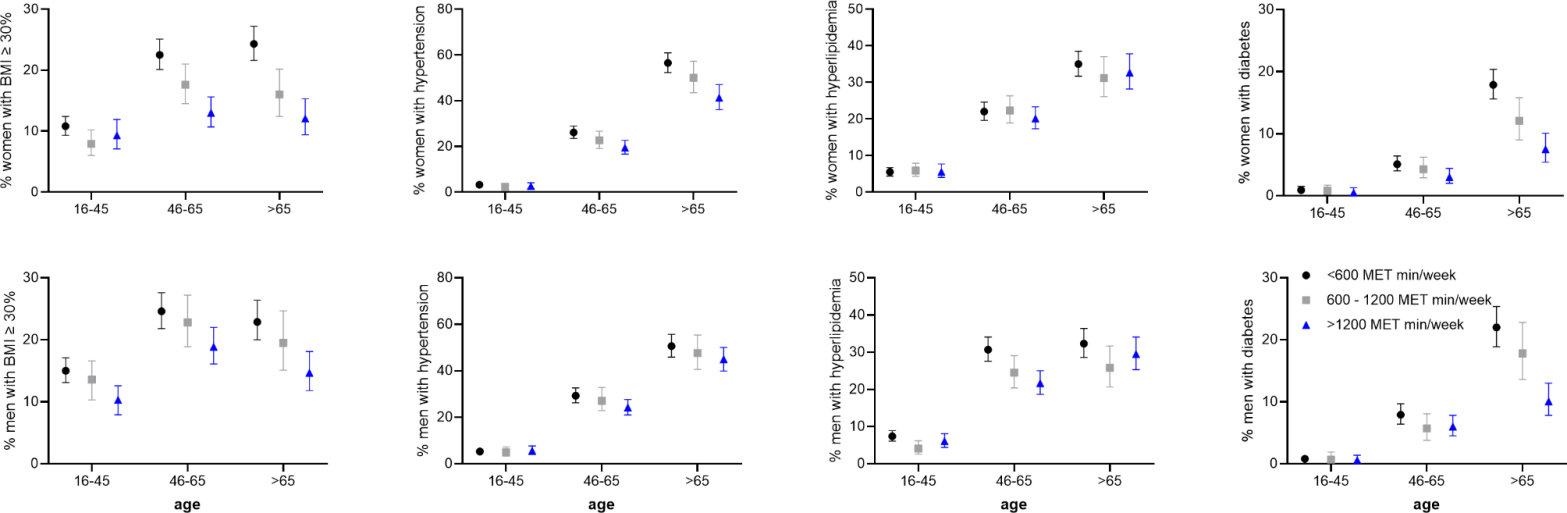
Sex- and physical activity-dependent prevalences (95% CI) of cardiovascular risk factors (obesity, hypertension, hypercholesterolemia, diabetes) for three age groups

The striking age effect on the prevalence of risk factors and its modulation by the PA level is graphically illustrated in Fig. 1. Notably, while the prevalence of obesity stabilized or even decreased above the age of 65 years, it steeply increased for T2D, especially in the lower PA categories. In contrast, the prevalence of hypercholesterolemia even increased in the group with the highest PA level.

Tables 4a and 4b show adjusted ORs (4a: for age, smoking, alcohol consumption, and dietary intake; and 4b: additionally for BMI) for the development of risk factors depending on the PA level (600–1200 MET min/week – meeting WHO recommendations – and > 1200 MET min/week as compared to < 600 MET min/week – not meeting WHO recommendations). The prevalence of obesity in women and of hypercholesterolemia in men was reduced in those who met (but did not exceed) WHO recommendations, but the sex differences for the prevalence of obesity disappeared when data were additionally adjusted for BMI (Table 4b). In contrast, exceeding WHO recommendations for regular PA reduced the prevalence of obesity and T2D by 31–55% and of hypertension by 15–33% in individuals of both sexes, while PA lowered hypercholesterolemia only in men (Table 4a). The beneficial effect of PA > 1200 MET min/week on hypertension disappeared in men when additionally adjusted for BMI (Table 4b).

**Table 4a:** Adjusted (for age, smoking, alcohol consumption, and dietary intake) odds ratios (95% CI) for the occurrence of cardiovascular risk in men and women as compared to the lowest level of physical activity (< 600 MET min/week)

Risk factor	600–1200 MET min/week	> 1200 MET min/week
	Men Women	Men Women
Hypertension	0.99 (0.84–1.17) 0.88 (0.75–1.04)	**0.85 (0.74**–**0.98) 0.67 (0.58**–**0.77)**
Hypercholesterolemia	**0.72 (0.61**–**0.80)** 0.98 (0.84–1.15)	**0.79 (0.69**–**0.91)** 0.98 (0.85–1.13)
Diabetes	0.88 (0.67–1.16) 0.79 (0.60–1.04)	**0.53 (0.42**–**0.67) 0.45 (0.34**–**0.58)**
Obesity	0.92 (0.84–1.09) **0.72 (0.61**–**0.85**)	**0.69 (0.59**–**0.79) 0.60 (0.51**–**0.70)**

**Table 4b:** Adjusted (for age, smoking, alcohol consumption, dietary intake, and BMI) odds ratios (95% CI) for the occurrence of cardiovascular risk in men and women compared to the lowest level of physical activity (< 600 MET min/week)

Risk factor	600–1200 MET min/week	> 1200 MET min/week
	Men Women	Men Women
Hypertension	1.03 (0.87–1.23) 0.93 (0.79–1.09)	0.92 (0.80–1.06) **0.72 (0.62**–**0.84)**
Hypercholesterolemia	**0.73 (0.61**–**0.87)** 0.99 (0.85–1.12)	**0.81 (0.71**–**0.94)** 1.02 (1.00–1.03)
Diabetes	0.91 (0.69–1.19) 0.83 (0.63–1.10)	**0.56 (0.45**–**0.71) 0.48 (0.37**–**0.64)**

## Discussion

Demonstrated PA levels of the Austrian population are higher than average percentages (34.8%) reported from the 27 EU member states [15] and similar or lower than those reported for older people (58–75 years) by a pan-European cohort study [16]. Although differences in estimates of insufficient PA may result from different measurement approaches, a lack of sufficient PA certainly exists in a considerable proportion of the Austrian population. Importantly, even smaller than recommended amounts of PA confer health benefits compared to inactivity [17]. In agreement with our findings, the above-mentioned reports also demonstrate consistently higher PA levels in men than in women, which may be due to a social desirability bias based on data that single-point assessment and different exercise identities can lead to overreporting in men [18]. Unexpectedly, the amount of reported weekly PA increased up to the age of 75 years in both sexes (Table 2). This may reflect an increase in PA after retirement [19] but could at least partly be due to self-reported PA overestimation due to a perceptional change of PA levels in older people [20]. In the present study, we were not able to evaluate a potential reduction in PA intensity (leading to lower MET min/week values) as age increased, which could be a further explanation.

On the one hand, we identified relatively small beneficial effects on the prevalence of main cardiovascular risk factors (obesity, hypertension, hypercholesterolemia, and T2D) in individuals who achieved PA levels corresponding to 600–1200 MET min/week (as recommended by the WHO) as compared to those who did not meet these recommendations. On the other hand, those who exceeded WHO recommendations (> 1200 MET min/week) displayed much more pronounced effects. These health benefits of PA become particularly important in older age groups, where the prevalence of risk factors is highest for both sexes (Table 3).

Similar benefits of elevated PA amounts on obesity and T2D may be associated with the relationship between these risk factors, as the prevalence of T2D is linearly and positively correlated with the increase in body mass [21]. Moreover, recent evidence suggests a bidirectional association between T2D and age-related sarcopenia, where people with sarcopenia have two-fold higher chances of having T2D [22]. Moreover, muscle mass and performance are lower in older patients with T2D than in their peers without T2D [23, 24]. Adding muscle-strengthening activities to aerobic PA on two or more days/week, therefore, is particularly recommended for older adults who have or are at risk of T2D and/or sarcopenia [25].

Higher PA levels may help to prevent people from gaining much weight by keeping them in a “regulated zone of energy balance”, where the intrinsic biological mechanisms that control the energy balance are optimized [26]. Generally, the beneficial effects of higher PA volumes may be related to substantial improvements in insulin sensitivity, probably regardless of exercise intensity and volume [27]. In the present study, individuals who exceeded the WHO PA recommendations (as opposed to those who did not meet these recommendations) displayed significant reductions in unadjusted obesity, and men and women older than 65 years of age displayed such reductions in T2D prevalence. Meeting these recommendations (600–1200 MET min/week) resulted in a reduced T2D prevalence only in women > 65 years (Table 3). The marked effect of relatively low PA amounts on T2D prevention observed in this study, and especially in women, is in line with findings for US adults [28]. On the one hand, equivalent doses of PA may be more effective in women than men as demonstrated for all-cause and cardiovascular mortality risk reduction [29]. On the other hand, unhealthier lifestyle factors (alcohol, smoking, nutritional habits) of men may partially explain the observed differences between the sexes seen in the current study, based on the observed loss of the gender gap in T2D prevalence in the PA category of 600–1200 MET min/week when adjusted for these factors (Table 4a).

A dose-response relationship between PA amounts and the risk of developing cardiovascular and metabolic diseases has been demonstrated repeatedly [7, 30–34]. In particular, short-term studies suggest that weight loss due to PA is related to total fat loss in a dose-dependent manner [30]. PA amounts exceeding WHO recommendations have been shown to be especially effective for reducing the prevalence of obesity and T2D [7]. A systematic review of longitudinal observations found lower incidences of obesity and T2D in those with high levels of regular PA [35]. Bell et al. reported that, compared to low PA, the incidence of obesity was 37% lower after a 10-year follow-up in those performing great amounts of PA [36], while Carlsson et al. showed a 44% reduced risk of T2D development (high vs. low PA) over an about 30-year observation period [37]. In line with our findings, a meta-analysis reported a T2D risk reduction of 26% (95% CI 20–31%) among those who achieved PA amounts of 150 min/week [32]. This risk was further reduced by 36% with PA amounts of 300 min/week, and even by 53% at even higher levels (60 MET h/week). Increasing moderate PA (150–300, 300–600, and > 600 min/week) in a UK Biobank cohort resulted in a comparable reduction in T2D risk of 49% (95%CI 62–32%), 62% (71–50%), and 71% (80–59%), respectively, as compared to those achieving moderate PA amounts < 150 min/week [7].

The results of the present study on the effects of PA on hypertension in Austrian people is partly in accordance with findings from other populations, demonstrating no PA effect in men [38], but a hypertension-risk reduction in women with PA amounts > 500 MET min/week [39]. A higher frequency and longer duration of moderate or vigorous PA was also associated with a reduced risk of developing systemic hypertension in a Chinese study [40]. Individuals of the highest PA volume quartiles (3rd and 4th ) showed reduced hypertension development by 18% (OR 0.82, 95% CI 0.72–0.95) and 22% (OR 0.78, 95% CI 0.68–0.91) [40].

Similarly, positive but less pronounced effects of PA on hypercholesterolemia have been previously reported [41]. Generally, PA beneficially modulates primarily HDL-C levels (increase) with smaller effects on LDL-C levels (no change or slight decrease) and total cholesterol levels [42]. Consequently, the present findings should be interpreted with caution, since changes in both HDL-C and LDL-C contribute to total cholesterol levels [41, 43]. According to a meta-analysis, a minimum PA volume of 120 min/week is necessary to increase HDL-C levels, and every 10-min PA prolongation is associated with an increase of about 1.4 mg/dL in HDL-C level [44]. This corresponds to an elevation of about 25 mg/dL HDL-C at a PA volume extrapolated to 300 min/week. Thus, based on our data, the steep increase in hypercholesterolemia in those who exceeded the WHO PA recommendations in the age groups of > 65 years may be a consequence of a more pronounced increase in HDL-C and a less notable decrease in LDL-C. This more favorable response of PA on HDL-C in older men agrees with the findings of a 12-week web-based intervention to increase PA in older adults [45].

### How to promote physical activity?

Time economy is a major obstacle to achieve sufficient amounts of PA. Accordingly, the 2018 US Physical Activity Guidelines for Americans suggest new opportunities for promoting PA by recognizing that even short and sporadic bouts of high relative intensity incidental PA already improve health [46]. Performing short bouts of PA is thus an attractive option for people living otherwise rather sedentary lifestyles, helping them to be more active and still reap PA-related health benefits.

Furthermore, urban environments with designs that promote PA provide opportunities to considerably influence people’s PA patterns. Thus, politicians who dare to take bold action and consider the health of their citizens and nations can influence PA levels by applying targeted architectural and infrastructure measures. For example, initiatives that encourage active (not motorized) commuting (e.g., by bicycle) could significantly reduce the burden of important chronic conditions [47].

Finally, the integration of PA promotion into primary, secondary, and integrated care settings for people with medical conditions could be a cost-effective way to improve the health of patients in Austria and elsewhere. Unfortunately, healthcare professionals and clinical staff often fail to encourage health-promoting behavioral change due to their limited educational background, which reduces their confidence in ‘prescribing’ PA. Therefore, a whole system approach that includes legislative measures to promote PA more effectively and develop educational resources (from undergraduate and postgraduate to continuing medical education levels) could effectively increase the capability, opportunity, and motivation of health care professionals to sustainably embed PA promotion into clinical practice at a system level [48].

### Limitations

The strengths of the present study include the large sample size, including more than 15,000 participants and representing the entire Austrian population. The main limitations arise from the use of self-reported data, e.g., a lack of uniform underlying diagnostic procedures and clear definitions for risk factors and age- and sex-dependent over- or underestimation (or -reporting) of body mass or PA volumes. A potential systematic shift in reporting behavior between the different age and activity groups cannot be excluded. Moreover, BMI alone cannot be used to differentiate between lean mass and fat mass or between subcutaneous and visceral fat. Furthermore, crucial parameters of PA such as intensity and duration were not reported. Thus, our exposure estimates differ from those derived using device-based measures, which also record light intensity and sporadic incidental vigorous PA events that are more difficult to recall [49]. Moreover, although this is a representative population-based cross-sectional survey, the sub-sample sizes—especially in the highest age groups—are too small to derive valid conclusions for these groups. Finally, the cross-sectional design of the analysis precludes temporal and causal conclusions regarding the associations between PA levels and cardiovascular risk factors. Therefore, reverse causation is a potential concern, and it cannot be ruled out that the true effects of physical activity on cardiovascular health are less pronounced than reported.

## Conclusions

The results of this population-based cross-sectional survey highlight the still-insufficient engagement in sufficient PA amounts (i.e., not meeting WHO recommendations) of people living in a central European country (Austria), and particularly the engagement of people in younger and older age groups. Men surveyed were more physically active than women but exhibited otherwise less healthy lifestyles. A reduced prevalence of obesity in women and of hypercholesterolemia in men was observed in people who reported meeting the WHO PA recommendations (600–1200 MET min/week). Large decreases in the prevalence of obesity and T2D (31–55% reduction) were observed in members of both sexes who exceeded the minimal WHO recommendations for PA (> 1200 MET min/week). These higher PA amounts seemed to reduce hypercholesterolemia only in men and systemic hypertension more robustly in women. The reported sex differences in PA levels and their association with cardiovascular risk factors may provide a basis for improved preventive health counseling. Although higher PA levels result in more beneficial health effects, already small amounts of PA are better than none.

## Data Availability

The datasets used for the current study are available free of charge and can be downloaded after registration and activation at: https://www.statistik.at/services/tools/services/amdc-mikrodaten-fuer-die-wissenschaft/scientific-use-files#c15273.

## Abbreviations

ATHIS: Austrian Health Interview Survey
BMI: Body mass index
HDL-C: High-density lipoprotein cholesterol
LDL-C: Low-density lipoprotein cholesterol
MET: Metabolic equivalent
PA: Physical activity
T2D: Type 2 diabetes mellitus
TG: Triglycerides
WHO: World Health Organization
